# Whole Genome Sequencing and Comparative Genomic Analysis Reveal Allelic Variations Unique to a Purple Colored Rice Landrace (*Oryza sativa* ssp. *indica* cv. Purpleputtu)

**DOI:** 10.3389/fpls.2019.00513

**Published:** 2019-05-07

**Authors:** V. B. Reddy Lachagari, Ravi Gupta, Sivarama Prasad Lekkala, Lakshmi Mahadevan, Boney Kuriakose, Navajeet Chakravartty, A. V. S. K. Mohan Katta, Sam Santhosh, Arjula R. Reddy, George Thomas

**Affiliations:** ^1^AgriGenome Labs Pvt. Ltd., Biotechnology Incubation Center, MN iHub, Genome Valley, Hyderabad, India; ^2^Medgenome Labs Ltd., Bengaluru, India; ^3^SciGenom Labs Pvt. Ltd., Cochin, India; ^4^SciGenom Research Foundation, Cheruthuruthy, India; ^5^AgriGenome Labs Pvt. Ltd., Kakkanad, India; ^6^Department of Plant Sciences, University of Hyderabad, Hyderabad, India

**Keywords:** Purpleputtu, rice, WGS, SNPs/INDELs, variant calling, anthocyanin pathway

## Abstract

Purpleputtu (*Oryza sativa* ssp. *indica* cv. Purpleputtu) is a unique rice landrace from southern India that exhibits predominantly purple color. This study reports the underlying genetic complexity of the trait, associated domestication and de-domestication processes during its coevolution with present day cultivars. Along-with genome level allelic variations in the entire gene repertoire associated with the purple, red coloration of grain and other plant parts. Comparative genomic analysis using ‘a panel of 108 rice lines’ revealed a total of 3,200,951 variants including 67,774 unique variations in Purpleputtu (PP) genome. Multiple sequence alignment uncovered a 14 bp deletion in *Rc* (*Red colored*, a transcription factor of *bHLH* class) locus of PP, a key regulatory gene of anthocyanin biosynthetic pathway. Interestingly, this deletion in *Rc* gene is a characteristic feature of the present-day white pericarped rice cultivars. Phylogenetic analysis of *Rc* locus revealed a distinct clade showing proximity to the progenitor species *Oryza rufipogon* and *O. nivara.* In addition, PP genome exhibits a well conserved 4.5 Mbp region on chromosome 5 that harbors several loci associated with domestication of rice. Further, PP showed 1,387 unique when SNPs compared to 3,023 lines of rice (SNP-Seek database). The results indicate that PP genome is rich in allelic diversity and can serve as an excellent resource for rice breeding for a variety of agronomically important traits such as disease resistance, enhanced nutritional values, stress tolerance, and protection from harmful UV-B rays.

## Introduction

Rice is the staple food for more than half of the world’s population and substantially meets both food and calorie requirements. Rice cultivation covers about 165 million hectares globally with an annual production of 758.8 million MT (Food and Agriculture Organization of the United Nations [FAOUN], 2017) and is a critical component of the global food security system. Diverse Asian population rely on rice to cover 35–80% of their calorie needs, while global reliance is about 21%. The two subspecies of cultivated rice, *O. sativa*, namely *indica* and *japonica*, occupy more than 90% of the Asian rice crop acreage (Muthayya et al., 2014). Evolution of rice from its progenitors is marked by great complexity as it appears to have involved diverse lineages, domestication/de-domestication processes and selection, both natural and artificial. Domestication and selection of different populations of Asian wild progenitor rices, *O. rufipogon* and *O. nivara* might have begun more than 10,000 years ago giving rise to the present day Asian cultivated rices (Yang et al., 2015; Choi et al., 2017; Qiu et al., 2017). Wild rices predominantly exhibit varying grain colors and this trait is known to be associated with domestication (Civáň and Brown, 2017). Rice germplasm collections comprise various colored rice lines, though these are neither cultivated widely nor used extensively in crop improvement programs. Colored rices have been widely used as entries in trials for the discovery of genes that confer resistance to bacteria, fungi and insects (Ahuja et al., 2010). Colored rices of various hues were described as red, brown, purple, and black, based largely on pericarp and/or hull coloration due to accumulation of anthocyanins, their precursors, flavonoids or their combinations, called co-pigmentation, besides other polyphenolic derivatives. Anthocyanins, the end products of anthocyanin pathway, are ubiquitous pigments known to be present in flowering plants. Naturally occurring rice landraces that accumulate anthocyanins, proanthocyanidins, and anthocyanin derivatives have been widely described (Reddy et al., 1995; Oh et al., 2018). Historically, colored rices have been deemed specialty rices by various ancient Asian cultures. For example, black rice has been described as forbidden rice or Emperor’s rice in China and red rices have been used in some religious celebrations in south and southeast Asia. However, due to changed consumer preference for white grained rices, they were not exploited in the breeding programs despite their special features such as enhanced levels of antioxidant compounds and biotic and abiotic stress tolerance (Reddy et al., 2007). In addition, red/purple rices exhibit some well described domestication related traits, though in varying intensity, such as seed dormancy, grain shattering, photo-period sensitivity, long duration, tillering, and lodging.

Purpleputtu (PP) is a colored landrace that exhibits purple color in all aerial parts including seeds except in nodes and pollen (Reddy et al., 1995). It is an *indica* landrace cultivated in small restricted areas in farmer fields in southern India, often used as border lines to demarcate test plots in experimental fields, primarily serving as a pollen barrier due to its height (Rangaswamy et al., 1988). The genetic control of pericarp color in PP has been described and molecular biological basis of the control of the underlying anthocyanin pathway has been elucidated (Reddy et al., 1994, 1995, 2007; Oh et al., 2018). Earlier studies on color in *japonica* rices revealed the contours of the genetic circuitry that govern color pathway (Furukawa et al., 2007). Regulation of the anthocyanin pathway, both in *indica* and *japonica* subspecies, by different classes of transcription factors and repressors have been identified and tissue specific expression of some of these genes deciphered (Reddy et al., 1995; Sweeney et al., 2006; Rahman et al., 2013).

Allelic variations at certain target loci of the anthocyanin pathway that lead to the formation of many diverse flavonoids and anthocyanins have been described (Reddy et al., 1995; Kim et al., 2011, 2015; Maeda et al., 2014; Chin et al., 2016). However, not much is known about allelic variations at loci associated with the pathway in terms of mutations, deletions and rearrangements. Scant information exists on differences at the genomic level between colored and white grained rices. Advancement of next generation sequencing (NGS) technologies along with the availability of the reference genome sequences for both *japonica* and *indica* rices provided an unprecedented opportunity to investigate the genome wide distribution of allelic variations that control complex pathways such as those that differentiate colored rices from white rice. Deep sequencing coupled with comparative genomic analysis using extensively sequenced diverse rice lines and SNP-Seek database provide a great opportunity to gain incisive insights into the genetic and molecular basis of a diverse array of traits. Further, genomic analysis of diverse genotypes such as wild progenitors, land races, cultivars and modern rices is expected to throw new light on domestication, selection sweeps of specific genomic regions and evolution of colorless grain phenotype. Present day colored rices, i.e., PP, had evolved from their colored progenitors such as *O. rufipogon* over thousands of years of cultivation, domestication and natural selection. It is interesting to investigate as to how such a complex trait governed by many genes across the genome has evolved and maintained even when selection is biased toward white grained rices in modern time. Interestingly, colored rices are known for traits associated with disease resistance and abiotic stress tolerance as demonstrated by numerous reports of introgression breeding via wide hybridization to essentially transfer useful genes from progenitors and wild relatives to present day cultivars, a form of de-domestication.

The present study is aimed at understanding the basis of existence and maintenance of PP, a fully colored rice, by whole genome deep sequencing and comparative genomics using a global collection of thousands of rice lines including progenitors such as *O. rufipogon, O. nivara*; and the reference genome of *Nipponbare* rice (Kawahara et al., 2013; Lachagari et al., 2019). We uncovered a significant number of genome-wide allelic variations in PP including those in genes associated with anthocyanin biosynthesis and genomic regions associated with the domestication-related genes controlling dormancy, seed shattering and diseases response. Additionally, we report here the discovery of unique alleles in genomic regions showing extreme conservation through evolution and thus representing selection sweeps. Besides, we identified numerous unique alleles at loci associated with major structural and regulatory genes of the pathways determining purple phenotype.

## Results

### Whole Genome Sequencing of PP and Comparison With Nipponbare Reference Genome

Whole genome shotgun sequencing of PP genomic DNA (80× coverage) on Illumina HiSeq 2000 platform yielded 43.47 GB of raw data that include a total of 430,403,016 paired end reads of 100 bp. More than 81.7% of the data exceeded Q30 Phred quality score. The quality score of read 1 (forward) and read 2 (reverse) are shown in Supplementary Figures S1A,B, respectively, while position-based quality of each nucleotide for read 1 and 2 is shown in Supplementary Figures S1C,D, respectively. Read based GC content estimates show that more than 36% of reads have less than 30% of GC content (Supplementary Figure S1E). The data was deposited in NCBI SRA database with an accession number PRJNA309223. The reads were aligned to the reference genome (Os-Nipponbare-Reference-IRGSP-1.0, MSU release 7) using BWA. Overall, 95.7% of the total reads were mapped covering 94% of the reference genome. The reads with mapping quality value ≥30 were retained for further analysis after removing duplicates. Out of total generated reads, 263,679,866 were aligned to the reference genome with an average of 49.5× read depth and 80.61% genome-wide coverage (Table 1 and Supplementary Figure S2A). Supplementary Figures S2B–D indicate percentage of aligned reads, chromosome-wise average read depth and coverage, respectively.

**Table 1:** Chromosome wise distribution and read statistics of Purpleputtu (PP) genome.

Chromosome	# of filtered aligned reads	Coverage (%)	Average sequencing depth
Chr1	32,649,648	83.98	52.79
Chr2	27,376,930	86.39	53.48
Chr3	28,942,255	89.53	55.75
Chr4	23,276,859	75.41	45.82
Chr5	22,110,609	85.62	51.83
Chr6	21,539,768	79.92	48.35
Chr7	19,578,596	78.28	46.23
Chr8	19,486,081	79.60	48.04
Chr9	16,519,610	80.51	50.22
Chr10	15,698,995	78.79	47.25
Chr11	17,323,226	72.15	41.86
Chr12	19,177,289	72.06	48.36
Total/average	263,679,866	80.61	49.47


### Annotation of Unmapped Reads of PP Genome

Assembled unmapped reads of PP was used for repeats masking using rice as reference model. A total of 2,124 genes were predicted, out of which 1,239 gene sequences were annotated based on UniProt, NCBI NR and Phytozome database (Supplementary Table S1). Of the 1,239 genes sequences, set of 70 and 1,069 have characterized and uncharacterized gene information respectively from homologous rice cultivar sequences. The remaining 76 sequences were annotated to the orthologous sequences of maize, wheat, purple false brome, cutgrasses, and sorghum. A set of 885 sequences did not find suitable match in any of the above-said databases, indicating that they are unique in PP genome and their functional domain information was extracted to find out the functions of gene which are unique to PP (Supplementary Table S1).

### Identification of Variants in PP Genome and Variant Desert Regions

**FIGURE 1:**
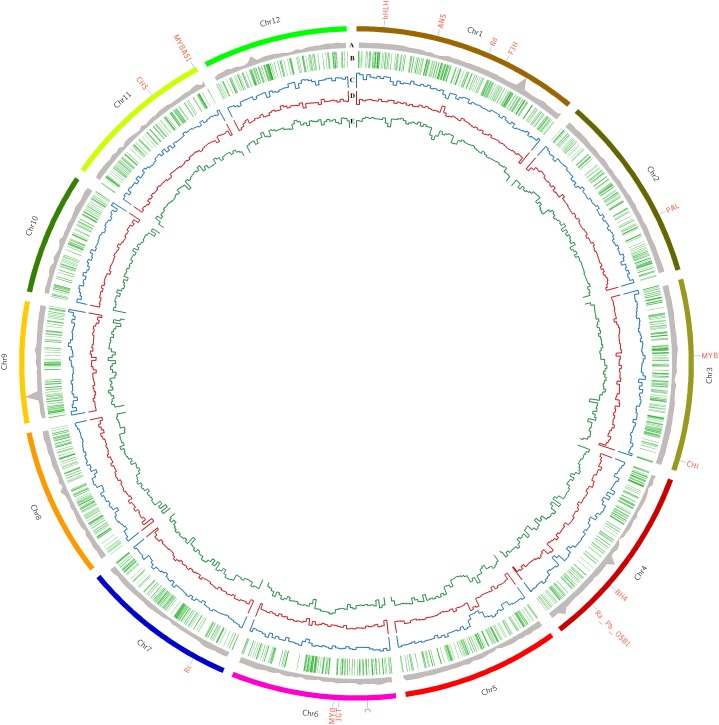
Comprehensive genome-wide analysis of Purpleputtu (PP) characteristics with Nipponbare reference genome. Circle diagram represents **(A)** read depth, **(B)** gene density, **(C)** insertion density, **(D)** deletion density, **(E)** SNP density. Outer circle of diagram represents the chromosomes of PP genome, each one labeled with important genes.

**FIGURE 2:**
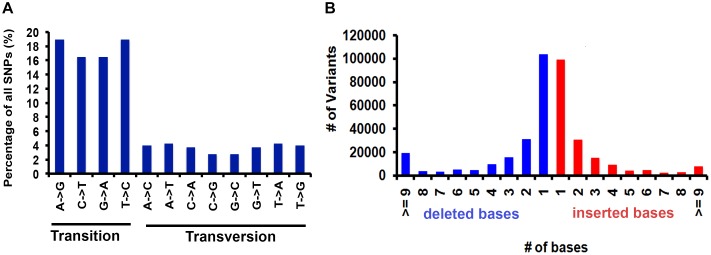
Base distribution statistics of variants identified in PP genome. **(A)** Percentage of transition and transversions. **(B)** Distribution of deleted and inserted bases.

Comprehensive genome-wide mapping diagram indicates the read depth, gene density, insertion density, deletion density, and SNP density (Figure 1). A total of 3,200,951 variants (2,824,513 SNPs and 376,445 INDELs) were identified in PP (Table 2) with read depth ≥5 and variant quality score ≥50 against *Nipponbare* reference genome (Supplementary Table S2). A majority of the variants (88.24% of SNPs and 94.44% of INDELs) were found to be homozygous. Most of the SNP changes observed were of transition type: A>G (18.92%), C>T (16.47%), G>A (16.43%), and T>C (18.89%) with a Ts/Tv ratio of 2.41 (Figure 2A). A majority (70%) of the identified changes are short INDELs of length 1–2 bp (Figure 2B). Of the total variants 1,058,815 (33.8%) map to the repeat region of the genome (Supplementary Table S3). The variant density was estimated to be 756 SNPs and 100 INDELs per 100 Kb in PP in comparison with MSU release 7 assembly. Chromosome (Chr) 10 shows the highest SNP density (889/100 Kb), while the lowest SNP density (624/100 Kb) was found in Chr5. Chr1 shows the highest INDEL density (109/100 Kb) whereas Chr4 has the lowest (85/100 Kb) (Supplementary Table S2). We observed that 67% of the variant desert region (Chr5) falls into repeat regions with a majority of the repeat class being putative retrotransposons as compared with a few other *indica* lines (Wang et al., 2009). The average read depth and coverage in this variant desert region was observed to be 43× and 81%. A total of 652 genes overlap were found in the variant desert region, half of which do not have a single variant. Of these genes 368 and 43 genes belong to the retrotransposon and transposon proteins, respectively (Supplementary Tables S4, S5).

**Table 2:** Summary statistics of various identified variants.

Total variants	3,200,951
Total SNPs	2,824,506 (88.24%)
Total INDELs	376,445 (11.76%)
Total homozygous variants	2,864,972
Total heterozygous variants	335,979
Transition/transversion (Ts/Tv)	2.41
Average read depth	51.95
Average variant quality	1707.037


### Annotation of Variants in PP Genome

**FIGURE 3:**
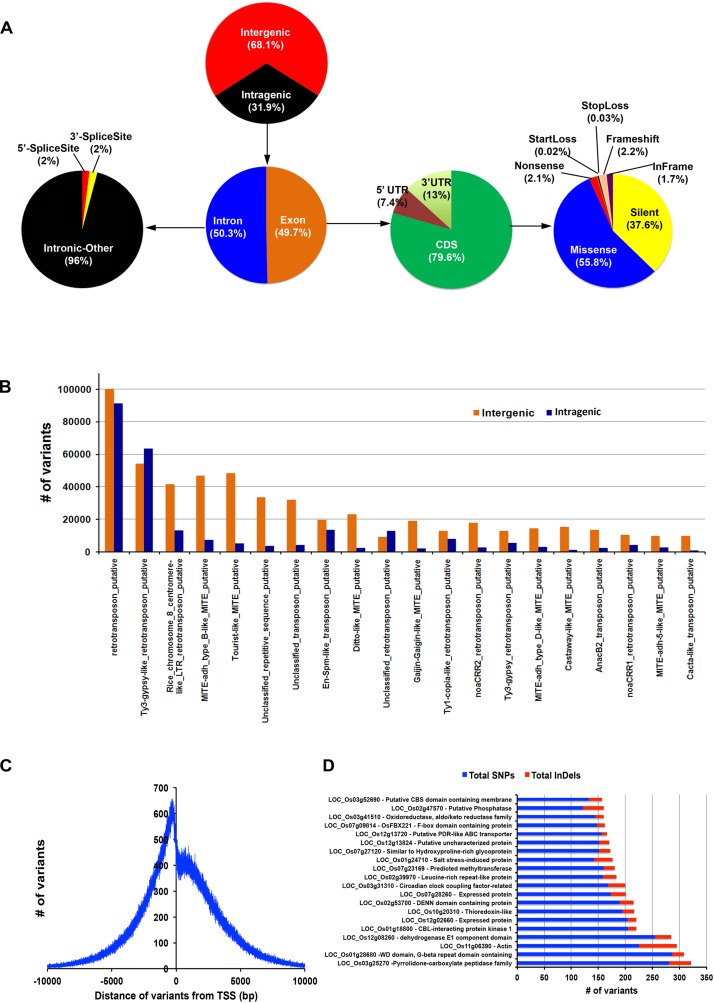
Comprehensive variant detection and annotation. Panel **(A)** indicates the distribution of variants in different regions of genome. Panel **(B)** represents the number of variant in different types of repeats. **(C)** Variant density graph from transcription start site (TSS). **(D)** Genes containing highest SNPs/INDELs variations.

The variants were annotated using an in-house pipeline against the gene model provided by MSU release 7. Overall, ∼32% of the variants span the genic region and the remaining 68% fall in the non-genic regions (Figure 3A). Out of the total genic variants, 50% overlap exonic region and of these 79.6% falls in the coding regions. Of the total variants in the coding regions, 37.6% were synonymous and the remaining 63.4% were non-synonymous type. About half (55.8%) of the non-synonymous type variants belong to missense class. We found that 33% of the total variants are in the repeat regions. Of these, 23.3% were genic and the remaining 76.7% were inter-genic. Further, a breakup of repeat class variants revealed that many of them belong to retrotransposons and few to miniature inverted-repeat transposable element (MITE) repeat class and cacta-like transposons (Figure 3B). These may serve as a valuable resource for selecting functional markers in genetic mapping programs. We further investigated the variant density around the transcription start sites (TSS) and found that it peaks at around 420 bp upstream and dips at ∼165 bp downstream (Figure 3C). The variant density gradually decreases toward zero on both sides of the TSS. Deep analysis of variants present in 1.5 Kb upstream shows that maximum variations were present in the genes for pyrrolidone-carboxylate peptidase, WD domain/G-beta repeat domain containing protein, actin, dehydrogenase E1 component domain containing protein and CBL-interacting protein kinase 1 (Figure 3D), all variants and associated genes are depicted in Supplementary Table S6.

### Unique Variants of PP Genome

To capture unique variants of PP, we compared its genome with a panel of 108 rice lines (Supplementary Table S7) covering different red rices, progenitors, landraces, modern cultivars spanning across sativa group (*indica*, temperate and tropical *japonica*) and Australian, aromatic and wild rices. This panel also includes other wild/progenitor species such as *O. longistaminata, O. brachyantha, O. barthii, O. meridionalis, O. nivara, O. glaberrima*, and *O. rufipogon*. A deeper analysis of the genome sequences with this panel allowed us to identify a set of unique alleles in PP genome. Out of 3,200,951 variants, 67,774 were found to be unique to PP (Supplementary Table S8). Of these, 64,394 are SNPs and 3,380 are INDELs, which include 390 INDELs that span exonic regions. Zygosity analysis of unique variants revealed that 49,025 (72.74%) are homozygous and 18,749 (27.66%) are heterozygous. Among these unique variants, 24,576 were mapped to genic regions and 43,198 to intergenic regions (Supplementary Table S8). The genic regions (12,831) span across 9,370 genes indicating that unique variations occur in almost one fifth of the total genes in rice. Further classification of unique variants in 5^′^ UTRs, 3^′^ UTRs, intronic regions and splice junction sequences shows 7,087 missense SNPs, 4,942 silent variations, 371 non-sense mutations, and 23 start-loss and 18 stop-loss variants. Analysis of SNP desert region of chromosome 5 revealed 224 unique variants of which 13 are INDELs and 211 SNPs (Supplementary Table S9). A set of 63 variants are uncovered in genic region of which 34 are silent mutations, 26 are missense mutations and one is a non-sense mutation. A majority of these variations are localized in Ty3/Gypsy class of retrotransposons. Variant analysis also identified 96 unique variants of PP genome associated with morphological traits, physiological traits and resistance or tolerance to biotic and abiotic stresses (Supplementary Table S10).

### Comparison of PP Genome With Global Collection of 3,023 Rice Lines

Purpleputtu genome was subjected to a deep comparative analysis by aligning with International Rice Genome Sequencing Project (IRGSP v1.0) assembly^1^ having 3K SNP-Seek data set. The resultant 1,962,843 SNPs in PP were compared with 5,854,680 SNPs of 3K global rice collection to identify rare variants with high effect. PP variants were merged with those of 3K SNP-Seek dataset and rare variants were obtained with minor allele frequency (MAF) cutoff of <0.01. The dendrogram made with 3K dataset comparison shows that PP is close to IRIS-313-8921, IRIS-313-8498 (Supplementary Figure S5). A total of 481,205 rare SNP loci were found amongst the combined dataset; which has 5,323,594 SNPs in common variant loci. Of all rare variants, 479,818 loci were only called in PP and not called in 3K dataset; therefore, they were denoted as unique. The remaining 1,387 SNP loci/SNPs were found to be unique to PP and identified one each of stop loss, stop gained and splice site accepter variants having high effects (Supplementary Table S11). The Armadillo-like helical domain-containing protein has a stop gained mutation in exon 2 leading to a truncated protein. However, the other two mutations were observed in two different conserved hypothetical proteins. The chromosome-wise distribution of these unique SNPs are as follows: Chr1 (141), Chr2 (100), Chr3 (100), Chr4 (129), Chr5 (108), Chr6 (124), Chr7 (114), Chr8 (132), Chr9 (102), Chr10 (101), Chr11 (128), Chr12 (108). The localization of these SNPs in their respective loci is listed in Supplementary Table S11.

### Functional Classification of Variants

**FIGURE 4:**
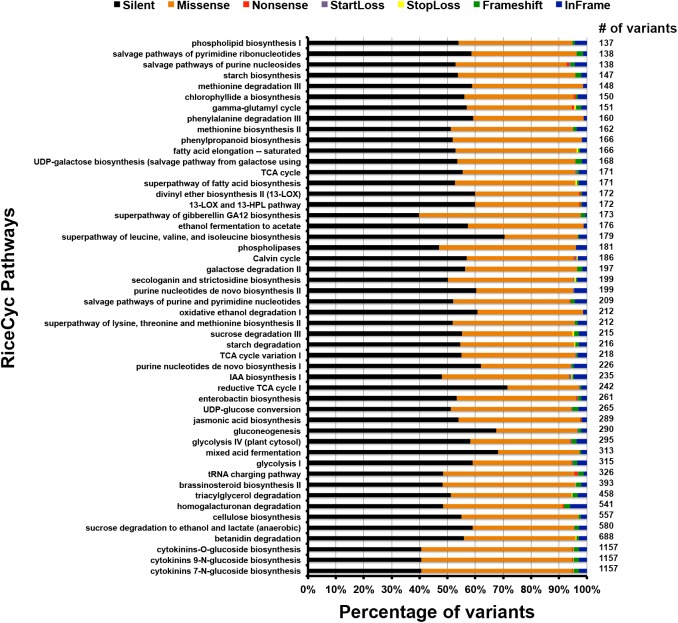
Bar diagram showing total number of different variants and their association in various metabolic pathways.

**FIGURE 5:**
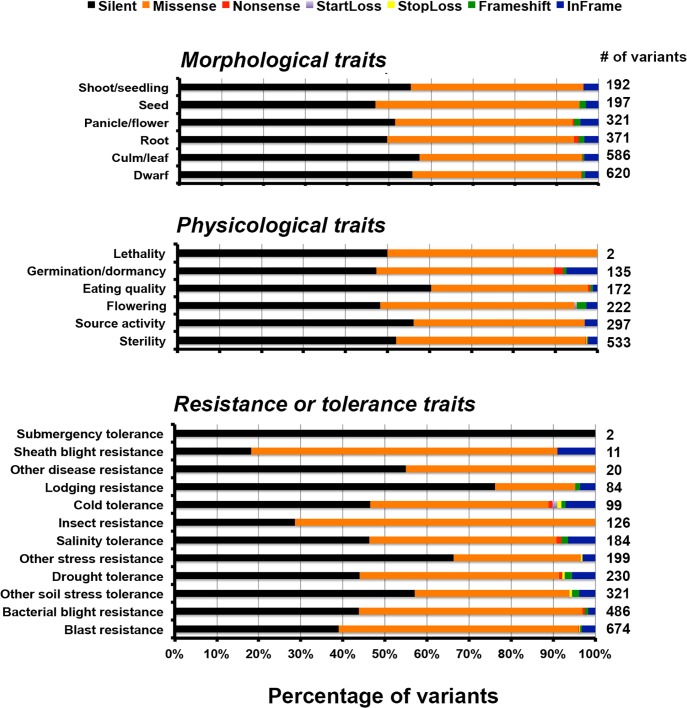
Bar diagram showing total number of different variants involved in morphological, physiological traits, and resistance to biotic and abiotic stress.

The functional classification of the variants was performed at different levels: pathway, ontology and traits. For the pathway study we compared the variants against rice metabolic pathway RiceCyc v3.3 database (Dharmawardhana et al., 2013). The pathways with the highest number of variants include cytokinins glucoside biosynthesis, betanidin degradation, sucrose degradation to ethanol and lactate, and cellulose biosynthesis (Supplementary Table S12) (Gupta et al., 2017a). Betanidin degradation eliminates betacyanin pigment pathway (which leads to production of red, purple, and violet betacyanin pigments which are predominant in *Caryophyllaceae*); here it is worth noting that anthocyanins and betacyanins are mutually exclusive in flowering plants (Rodriguez-Amaya, 2018). Similarly, sucrose degradation is a prerequisite for preventing root hypoxia. There is some information on the role of this process in salinity tolerance (Behr et al., 2017). Cytokinin glucoside biosynthesis is reported to be associated with indeterminate growth. Of the total unique variants in PP, 25,447 variants mapped to genes associated with 338 pathways (Figure 4). Of these, 13,439 are silent, 10,871 are missense, 66 are non-sense, 26 are start-loss, and 43 are stop-loss mutations. Interestingly, PP exhibits many unique variations at diverse loci controlling the highly conserved ubiquitous flavonoid biosynthetic pathway. These variants span across genes controlling sub-pathways such as flavonoid biosynthesis (PWY1F-FLAVSYN), flavonol biosynthesis (PWY-3101), anthocyanin biosynthesis [pelargonidin 3-*O*-glucoside, cyanidin 3-*O*-glucoside] (PWY-5125), anthocyanin biosynthesis [delphinidin 3-*O*-glucoside] (PWY-5153), proanthocyanidin biosynthesis from flavanols (PWY-641). Besides this, other biotic and abiotic stress responsive pathways were also found to harbor unique variants in PP (Supplementary Table S11) (Gupta et al., 2017a,b, 2018a). Specifically, 122 variations were identified in flavonoid biosynthesis pathway (PWY1F-FLAVSYN) genes of which 62 are silent and 57 are missense variants, 1 each of non-sense, frame-shift and in-frame variations. As many as 40 variants were observed in the flavonol biosynthetic pathway (PWY-3101). Considering the pathway-based analysis, it is inferred that pelargonidin/cyanidin 3-*O*-glucoside sub-pathway (PWY-5125) consists of 34 variations in which 9 are silent, 23 are missense, 1 each of frame-shift and in frame variations. In contrast, 24 variations were observed in an evolutionarily silenced delphinidin route of anthocyanin biosynthesis pathway (PWY-5153) of rice. Only one missense mutation was observed in enzymes responsible for proanthocyanidin pathway from flavanols (PWY-641) in rice. Trait ontology analysis for unique variants in PP was performed using Q-TARO database (Yonemaru et al., 2010). Out of this 67,774 variants, 6,283 were mapped to the regions associated with morphological (2,287), physiological (1,361), resistance or tolerance traits (2,436) and other traits (199) (Supplementary Table S13). Among these 2,812, 2, and 8 variants were found to be missense, start-loss and stop-loss, respectively. Interestingly, the start-loss variations are observed only in flowering and cold tolerance traits whereas stop-loss variants were observed in genomic locations associated with sterility, and various biotic and abiotic stress traits such as blast resistance, soil stress tolerance, drought tolerance and cold tolerance (Gupta et al., 2017b). Of all the variants that mapped to the trait-associated loci, the highest number of variants mapped to dwarf, sterility and blast resistance categories (Figure 5 and Supplementary Table S13). In addition, variants were observed in traits related to seed, leaf, flowering, germination, drought tolerance, stress tolerance, and lodging resistance which reflect the typical phenotype of PP.

### Variations in Transcription Factor Genes

The unique variants of PP mapped to different transcription factors (TFs) in rice including *MYB, bHLH, FAR1, NAC, WRKY, ERF, bZIP*, and *AP2* (Supplementary Table S14). The highest number of variants (1,752) was localized in Far-Red Impaired Response 1 (*FAR1*) family transcription factors which controls the far-red light signaling pathway by modulating phyA expression by activating *FHY1* and *FHL*, thus maintaining homeostasis (Lin et al., 2007; Gupta et al., 2019). In contrast, whirly family of transcription factors involved in defense response show low number of variants, viz., three, indicating high level of conservation. *NAC* transcription factor family, which is one of the largest families of plant-specific transcription factors and play an important role in response to various plant stresses show 682 variants (Nakashima et al., 2012). The *bHLH* class of transcription factors, including the *Rc* (red color), which are involved in flavonoid/anthocyanin biosynthesis besides other biological processes such as wound and drought stress, light signaling and hormone signaling shows 672 variants (Carretero-Paulet et al., 2010). Interestingly, 319 of them are missense, 3 non-sense, 15 frame-shift and 79 in-frame mutations and the remaining are silent; all of these changes put together indicate significant variation in this locus. *MYB* genes and related genes of rice, known to be involved in the regulation of anthocyanin biosynthetic pathways as well as in other different biological processes in rice were found to have 898 variants of which 433 are missense variants (Ambawat et al., 2013; Liu et al., 2015). The *WRKY* class of transcriptional factors show 483 variants of which 157 are silent and 272 are missense mutations, which are involved in several biotic and abiotic stress responses (Phukan et al., 2016; Gupta et al., 2018b). A set of 47 missense variations and one stop-loss mutation is observed in AP2 transcription factor family genes which are involved in abiotic stress response in rice (Fu and Xue, 2010).

### Evolutionary Analysis of Bh4 Domestication Genes

We studied independent events of domestication in the PP genome, which can offer an extremely useful system for studying the genetic basis of parallel evolution with other rice genomes. A significant trait altered by rice domestication and de-domestication is hull color. Wild progenitors of two cultivated rice species have predominantly black-colored hulls (*Bh4*) and straw colored hulls; these are the phenotypic effects of *Bh4* and straw hull 4 (*Sh4*) candidate gene expression (Zhu et al., 2011; Vigueira et al., 2013). Examination of evolutionary relationship of *Bh4* genes of PP with other 70 rice cultivar genes is shown in Supplementary Figure S3. The phylogenetic tree shows the *Bh4* DNA sequence variations, that provides clues to the parallel evolution of hull color variation in PP and other rice cultivars. Supplementary Figure S3 shows that out of 70 rice cultivars, 19 have variations suggesting that the same gene is responsible for parallel trait evolution.

### Variations in Anthocyanin Pathway Genes

The variants that mapped to the anthocyanin pathway genes were analyzed for the resultant changes in the encoded proteins and these include both regulatory and structural genes such as *C* (Chr6), *Ra* (Chr4), *Rc* (Chr7), *bHLH* (Chr1), *Rd* (Chr1), *MYB* (Chr3), *MYB* (Chr6), *MYBAS1* (Chr11), *chalcone synthase* (*CHS*) (Chr11), *chalcone isomerase* (*CHI*) (Chr3), *leucoanthocyanidin dioxygenase* (*ANS*) (Chr1), *flavanone 3-hydroxylase* (*F3H*) (Chr1), *3-O-glucosyltransferase* (*3GT*) (Chr6), *Bh4* (Chr4) and *phenylalanine ammonia-lyase* (*PAL*) (Chr2) (Figure 1). The variants of anthocyanin pathway genes were explored to understand the key differences that may explain the uniqueness of PP genome in showing distinct color phenotype (Cingolani et al., 2012). Of the 585 variants observed, 388 were identified as upstream variants followed by intronic (99), missense (45), synonymous (37) and 1 stop-gain variant (Supplementary Table S15). Comparative analysis of 5 cM Rc locus (Chr7) spanning 42.6 cM to 47.7 cM in three BAC clones (AP003748, AP005098, AP005779) revealed unique variations in PP placing it as a distinct clade within the *indica* group, separated from all other rice lines (Sweeney et al., 2006). Contrary to expectations, PP with its colored pericarp phenotype shows the 14 bp deletion signature in *Rc* gene which is a characteristic feature of the present white pericarped rice lines. Further evolutionary relationship of PP *Rc* gene to other *Rc* gene homologs of rice cultivars (includes *Australian, Elite, Indica, Tropical Japonica, Aromatic, O. barthii, O. glaberrima, O. rufipogon, O. meridionalis, O. longistaminata, O. brachyantha*, and wild species, i.e., BHA-Redrice, CA97_053, CA97_053, Daldhal, HK47, MV98_80, Nivara-IRGC106154, SHA-Redrice, IRGC105327, P46, VOC4, Yuan3_4) indicate divergence from other rice cultivars (Supplementary Figure S4). MYBAS1 gene (LOC_Os11g47460) on Chr11 was found to have 56 missense SNPs responsible for changes in 42 amino acids, which could be a possible paralog of the *Rc* gene on Chr7. In addition to that, the *Rc* gene (LOC_Os07g11020) on Chr7 exhibits an intronic variation responsible for deletion of ‘GAGA’ at position 138 that does not affect the functions of the gene. In addition, homology search for *Rc* genes against other rice genomes, did not provide any significant hit for any alternate functional gene. Notably, anthocyanin regulatory gene *Ra* (Pb/OSB1/LOC_Os04g47080) on Chr4 which is reported to be associated with purple color pericarp (Wang and Shu, 2007) shows one missense variation leading to M64T, and two frame shift variations causing T575fs and V545fs. Both of these frameshift mutations in this gene lead to alternate chain of 30 amino acids (PLGAGINIGWSPWTDTS QVCLICCRRTWE^∗^) in the C terminus when compared to *Nipponbare* reference genome. *bHLH* (LOC_Os01g09900) on Chr1 shows interesting variations leading to amino acid changes (E304K, P148L, V76A and disruptive in-frame insertion at A98AA). *MYB* gene (LOC_Os06g19980) on Chr3 shows a total of 14 variations in which 7, 3, 2, and 1 are downstream gene variant, in-frame deletion, intron variant and synonymous variant, respectively; remaining 1 variant belongs to missense variation and the changes in amino acid A263G possibly does not alter the secondary structure of the protein. *MYB* genes (LOC_Os06g19980) on Chr6 has 23 missense variations causing changes in amino acids, *viz.* S27R, M47L, R63L, R66L, D72G, L80N, I82S, A83P, I86V, Q112E, S116I, E147G, E148D, I151V, L163V, T202A, I232T, L296R, R298G, S446L, Y607H, and A639P; these changes possibly alter the structure and function of this *MYB* protein. The gene encoding PAL (LOC_Os02g41630) enzyme on Chr2 catalyzing the formation of 4-coumaroyl-CoA from phenylalanine has one synonymous, one intronic variation and only one missense variation changing amino acid A621V of the protein. *CHS* (Os11g0530600) on Chr11 has 1 SNP leading to a single amino acid change, N158S. *F3H* (LOC_Os01g50490) on Chr1, the gene encoding the enzyme involved in conversion of flavonone to dihydroflavonones has 49 unique variations in which 22 SNPs lead to protein structure variations. We found four SNPs each causing amino acid changes to proline and glutamic acid, *viz*. T3P, A79P, L99P, L474P, and Q80E, K407E, D410E, D414E, respectively. In addition, the remaining SNPs cause mutations of L6V, T15M, V34A, S55G, N77T, D103N, A110V, C389S, L392F, I393V, R418G, G420D, G424W, and T488A. Possibly, these mutations contribute toward hyper-accumulation of anthocyanins in PP. One silent variation was identified in *ANS1* (LOC_Os01g27490) gene on Chr1, encoding the key enzyme involved in the conversion of leucoanthocyanidins to anthocyanidins, the penultimate step in anthocyanin biosynthesis. The last enzyme 3*GT* (LOC_Os06g18790) of this pathway was found on Chr6 of PP genome, converting anthocyanidins to anthocyanins and had three unique SNPs (M186V, K190Q, K190R) that may have a role in accumulation of pigments in PP (Brazier-Hicks et al., 2007).

## Discussion

*Oryza sativa*, an independently domesticated rice that has been in cultivation for more than 12,000 years, has become the predominant cereal staple for most of the ∼3 billion plus Asians. With an estimated 30% rise in population by 2050 and unpredictable climate changes, rice breeders must gear up for substantial increase in rice productivity across various agroclimatic regions. Modern rice breeding technologies are increasingly utilizing the genetic resources of progenitors and wild relatives by introgression of new genes associated with important agronomical traits into cultivars. These mainly include genes for biotic and abiotic stress tolerance, growth, and maturity traits. Though colored rices constitute a significant proportion of germplasm collections, they were not extensively used as genetic resources in breeding. Progenitors and domesticated colored rices were reported to be potential source of genes for resistance to diseases and pest, and tolerance to abiotic stresses. Besides, diverse colored pigments are recognized for their nutritional quality and anti-oxidant properties. Further, colored rices were found to be good subject material for understanding domestication and de-domestication processes in grasses in general and rice in particular (Choi et al., 2017; Qiu et al., 2017). We set out to uncover novel and unique alleles in one such fully colored rice line PP, that is cultivated sporadically with no evidence of any directional selection or crossing in its long history of domestication and cultivation.

Whole genome sequencing of PP rice and mapping with Nipponbare genome was performed to discover genome-wide DNA variations. A total of 263,679,866 reads were mapped to the reference genome. The assembly and annotation of unmapped reads indicates the presence of many uncharacterized genes having homology with the other rice species such as *O. nivara, O. meridionalis, O. glumipatula, O. glaberrima, O. brachyantha, O. barthii, O. punctata, O. rufipogon*, and *O. alta* (Supplementary Table S1). Interestingly, 56 gene models showed homology with a red rice line (*O. punctata)* and all these genes are uncharacterized indicating the presence of novel genes associated with red/purple pigmentation. Further, comparative genomic analysis using ‘a panel of 108 rice lines’ spanning both *indica* and *japonica*, progenitors, land races uncovered 64,349 SNPs and 3,380 INDELs unique to PP genome. In all, we captured unique variants in one fifth of the total gene models of rice. Of the 3,200,951 polymorphic SNPs identified, about a third span across exonic regions and three fourth of them fall in coding sequences. Further, about 33% of the variants are mapped to repeat regions, retrotransposons and transposable elements, a finding that falls within the range reported for *Oryza* (Stein et al., 2018). The present data show that Chr1 has the highest number of INDELs and Chr10 has the highest number of SNPs per 100 kb. Similarly, the lowest INDEL density was on Chr4 and lowest SNP density on Chr5. In addition, distribution of SNP-rich and SNP-poor regions in each chromosome of PP was identified, which also corroborates with earlier findings in rice, Arabidopsis, and wheat (Nordborg et al., 2005; Ravel et al., 2006; Subbaiyan et al., 2012).

The mapped genome of PP revealed a clear bias toward transitions (almost twice that of transversions) deviating from the expected ratio of 0.5. Higher Ts/Tv ratios were reported in rice, maize, otus, medicago, diploid wheat, *Triticum monococcum* and barley (Batley et al., 2003a; Vitte and Bennetzen, 2006; Subbaiyan et al., 2012; Bindusree et al., 2017). Due to wobble effect, transitions manifest mostly into silent mutations that do not alter the amino acid and thus conserves the amino acid chain (Wakeley, 1996). Among transitions, the C/T transitions were more in number, presumably due to a simple methylation being the cause of this mutation (Coulondre et al., 1978). Higher frequency of C/T mutations has been reported in other crops such as common bean, maize, grape, and citrus (Batley et al., 2003b; Lijavetzky et al., 2007; Terol et al., 2008). Generally, genomic segments with higher SNP frequency were shorter than the lower SNP frequency segments. The SNP-poor region having 4.3 Mb between 9.3 Mb and 13.6 Mb on Chr5 was identified in PP genome that is popularly described as ‘SNP desert’ and described in detail (Supplementary Tables S4, S5, S9). This conserved region in rice has been earlier reported in certain *indica* and *japonica* lines (Wang et al., 2009; Yonemaru et al., 2010; Subbaiyan et al., 2012). It is well known that selective sweeps during the long process of domestication in both *indica* and *japonica* rice are responsible for lower SNP frequencies across some regions in the genome (Subbaiyan et al., 2012).

The progenitors of the present-day rice lines and most of the wild rices exhibit red color in the pericarp and some other plant parts. However, the present-day modern rice cultivars predominantly have white pericarp. The disappearance of purple–red–black–brown color in grain in modern cultivars is a great example of agronomical spreading of a single variation at the *Rc* locus through the course of domestication and natural and artificial selection (Furukawa et al., 2007; Sweeney et al., 2006; Gross and Olsen, 2010). The *Rc* locus of PP shows the well characterized 14 bp deletion in the fifth exon; this deletion has been reported to be the hallmark of white rices, yet the pericarp of PP that bears this signature, is colored. In contrast, almost all white pericarped lines, including all modern rice cultivars, have the same deletion at *Rc* locus. The *Rc* gene encodes a *bHLH* TF that regulates *DFR* activity. We predict an alternate TF that may be regulating *DFR* in PP grain. It is to be noted that the *Rc* locus in the colored progenitor *rufipogon* lacks this 14 bp deletion. It may be the result of gene flow from cultivars to PP which indicates de-domestication. Introduction of many disease resistance genes, such as *Xa21*, from wild progenitors by introgression breeding and directed selection is a routine approach to exploit novel genes. We conclude that the *Rc* locus of PP is conserved due to simple cultivation and non-directional selection for color. The white pericarp phenotype controlled by the deletion-containing *Rc* locus in modern rice cultivars is due to selection pressure for white grain. Our results challenge the notion that the 14 bp deletion is an invariant signature of white rices only.

In depth analysis of variants revealed several unique SNPs/INDELs in structural and regulatory genes of various pathways responsible for stress response, genotypic and phenotypic effects on different traits of PP rice (Supplementary Tables S12, S13 and Figure 4). Loci associated with cytokinin glucoside biosynthesis, betanidin degradation, sucrose degradation to ethanol and lactate, and cellulose showed the highest number of variants. Furthermore, our results revealed unique variations at loci associated with biosynthesis of betacyanin, anthocyanins, and flavonoids. It is well known that betanidin degradation is a prerequisite step in eliminating betacyanin production in plants. It is worth noting that most flowering plants do not accumulate betacyanins and thus these two different color pigments are mutually exclusive (Shoeva et al., 2016; Khoo et al., 2017; Rodriguez-Amaya, 2018). Sucrose degradation is a prerequisite for preventing root hypoxia and salinity tolerance in some plants. Cytokinin glucoside biosynthesis is reported to be associated with indeterminate growth. The unique variant analysis uncovered 25,447 variants mapped to different genes associated with 338 pathways (Figure 4). Many structural, regulatory and inhibitory genes (Table 3) dispersed across the genome are involved in the anthocyanin pathway that leads to purple color formation in various tissues in PP (Reddy et al., 1995). The production of anthocyanins such as cyanidin, pelargonidin, and delphinidin derivatives through a multistep anthocyanins pathway is mediated by several enzymes *PAL, cinnamate 4-hydroxylase* (*C4H*), *4-coumarate CoA ligase* (*4CL*), *CHS, CHI, F3H, dihydroxyflavonol reductase* (*DFR*); *leucoanthocyanidin dioxygenase* (*LDOX)*/*anthocyanidin synthase* (*ANS*), *GT*/*3GT, acyltransferase* (*AT*), methyltransferase (*MT*) (Aza-González et al., 2012). We found several unique variations in genes encoding *PAL, CHS, F3H, ANS*, and *3GT* enzymes of this pathway in PP when compared with ‘a panel of 108 rice lines’ and ‘SNP-Seek dataset.’ Variation (A621V) was found in alpha helical region of the *PAL* enzyme that is positioned far away from the catalytic site, indicating unlikely effect on structural and functional role of this enzyme (Bata et al., 2018). Changes of N158S in *CHS* enzyme in PP when compared with *CHS* crystal structure of *Freesia hybrida* (PDB ID:4WUM) indicated change of charged side change amino acid N to uncharged side chain amino acid S at a location near the catalytic residues in PP *CHS*. This change may be enhancing the catalytic activity of the enzyme (Sun et al., 2015). The hydroxylation pattern of flavonoids controls their color, stability, and antioxidant capacity. We identified the gene encoding *F3H* in PP having 22 unique mutations, when compared with 3K dataset of rice, which may be relevant in the maintenance of purple color through generations. These may also be relevant for stability, antioxidant activity and stress defense capacity (Liu et al., 2014). Furthermore, analysis of structural and functional differences of PP *F3H* enzyme with that of white rice and other related cultivars indicates that it belongs to the plant *Cytochrome P450* gene family (Schoenbohm et al., 2000). Similarly, several silent mutations were identified in the *Ans* gene encoding *ANS1* enzyme that catalyzes the penultimate step of the anthocyanin pathway, namely conversion of leucoanthocyanidin to anthocyanidin (Reddy et al., 1996, 2007).

**Table 3:** List of structural, regulatory, and inhibitory genes involved anthocyanin pathway with their phenotypic effects.

Key anthocyanin genes	Phenotypic effect
**Structural**	

***C*** (chromogen)	Responsible for anthocyanin production; with an allelic series of *CB, CBr, C+* (null)
***A*** (activator)	Activation of C gene; essential for anthocyanin: with an allelic series of *AS, AE, A, A+* (null)
***Rc*** (brown pericarp)	Synthesis of pigments in pericarp
***Rd*** (brown pericarp)	Synthesis of pigments in pericarp

**Regulatory**	

***P*** (purple)	Distributor of anthocyanin pigments in the apiculus: alleles *P, PK, P+* (null)
***Pl*** (purple leaf)	Localizer of anthocyanin in leaf: alleles *Plw* (leaf blade, leaf sheath, auricles, ligule, and pericarp); *Pl* (leaf blade, leaf sheath, collar, auricles, ligule, node, and internode); *Pli* (leaf blade, leaf sheath, ligule, and internode); *Pl+* (null allele resulting into color less phenotype of tissue).
***Pn*** (purple node)	Localizer of anthocyanin in the node
***Prp*** (purple pericarp)	Localizer of anthocyanin in the pericarp

**Inhibitory**	

***I-Pl*** (inhibitor of purple leaf)	Inhibit action of both *Plw* and *Pli* alleles
***I-Pl1, I-Pl2, I-Pl3***	Inhibit action of the *Prp* locus
***I-Pl4, I-Pl5***	Inhibits action of *Pli* allele
***I-Pl6***	Inhibits leaf blade pigmentation
***Ilb***	Inhibitor of purple leaf


Recently, Sun et al. (2018) proposed a *C-S-A* gene model for rice hull pigmentation: *C1* that encodes a *MYB* transcription factor and acts as a color-producing gene, and *S1* that encodes a *bHLH* protein that functions in a tissue-specific manner. *C1* interacts with *S1* and activates expression of *A1*, which encodes a dihydroflavonol reductase (Sun et al., 2018). We uncovered unique variants of various TFs such as *MYB, bHLH, FAR1, NAC, WRKY, ERF, bZIP, bHLH, AP2* and others (Supplementary Table S14) responsible for different biological, molecular and cellular processes in PP. The role of *bHLH, MYB, MYBAS1* TFs and other genes such *F3H, Ra*, and *Rc* in regulating anthocyanins and other associated pathways responsible for PP color and texture (Supplementary Table S15) (Pireyre and Burow, 2015) has been described here. In addition, as many as 96 novel variations in PP rice with known effects on morphological, physiological effects and stress response phenotypes are also reported.

Overall present study deals with whole-genome variations in PP rice examined by identifying SNP and INDEL polymorphisms using ‘a panel of 108 rice lines’ and ‘SNP-Seek dataset.’ Base substitutions and distribution of DNA polymorphism over PP genome provides important insights into the molecular basis underlying phenotypic traits exhibited by the genotype. Further, the unique allele variations in different genes participating in flavonoid biosynthesis and anthocyanin pathways responsible for purple color in PP genome were revealed by comparative analysis. Unique SNPs identified in anthocyanin pathway occurring in both structural genes and regulatory transcription factors, will help to breed rice with high nutraceutical content, particularly, flavonoids that have antioxidant activity.

## Materials and Methods

### Plant Sample Preparation and Sequencing

Seeds of PP was germinated, and 15-day-old seedlings were used for genomic DNA extraction using Qiagen DNeasy kit (Qiagen). Qubit 2.0 fluorometer (Thermo Fisher) was used to quantify and NanoDrop 2000 (Thermo Fisher) for quality check of the isolated DNA. The DNA was fragmented to 300 bp size using a Covaris M220 focused ultrasonicator. The fragmented DNA was purified, and sequencing libraries were prepared using Illumina TruSeq DNA sample prep kit (Illumina Inc., United States) as per manufacturer’s specifications. The quantity and size distribution of the libraries were carried out using a Bioanalyzer 2100 (Agilent Technologies). The quantified libraries were subjected for whole genome sequencing on Illumina HiSeq-2000 platform (Illumina Technologies) by paired-end sequencing to generate 90-base pair long, small reads with an insert size of 200–350 bp (Supplementary Figure S2). Standard Illumina pipeline was used to filter the whole genome sequencing data. To remove low-quality reads and reads containing adaptor/primer contamination, FASTQ files were further subjected to stringent quality control using NGS QC Toolkit (v2.3) (Patel and Jain, 2012).

### Mapping of PP Genome

BWA software (v0.7) was used for the mapping of high-quality filtered reads against reference genome reference genome (Os-Nipponbare-Reference-IRGSP-1.0, MSU release 7) download from Rice Genome Annotation Project Database^2^ (Li and Durbin, 2009; Kawahara et al., 2013). Further, only uniquely aligned reads (with mapping quality ≥30 and minimum read depth 10) were considered in this analysis. Base quality score re-calibration and INDEL realignment were performed using Genome Analysis Toolkit (GATK, v2.1.13) and genome-wide coverage was estimated by Samtools (v0.1.16) (Li et al., 2009; McKenna et al., 2010).

### *De novo* Assembly of Unmapped PP Genome and Functional Annotation of Genes

Unmapped reads of PP genome were assembled using MaSuRCA (v3.2.1) assembler with default options (Zimin et al., 2013). The assembled genome was masked using RepeatMasker (v4.0.6) with default parameters, using rice as a model (Smit et al., 2015). Subsequently the masked genome was used in Augustus v3.2.1 using rice as model organism for gene prediction (Stanke et al., 2008). The annotations of identified genes were done using the Diamond program against NCBI NR, UniProt and Phytozome (v11.0) databases (Buchfink et al., 2015). Also, domain based functional annotation of all genes was performed using InterProScan v5.33.72 (Mitchell et al., 2018).

### Identification and Analysis of Variants in PP Genome

Minimum variant frequency of ≥90%, average base quality of the SNP ≥30 and minimum read depth of 10 were the stringent criteria followed to filter the identified SNPs and INDELs. If three or more SNPs were present in any 10-bp window, the SNPs and INDELs were filtered. Genome wide distribution of DNA polymorphisms was analyzed by calculating their frequency at every 100 Kb interval on each rice chromosome. Circos was used to visualize the distribution of the SNPs and INDELs on rice chromosomes (Krzywinski et al., 2009). Such distribution is assessed by integrating the position of DNA polymorphisms with GFF file containing rice genomic annotation. Customized Perl scripts were used to perform the genomic distribution and annotation of SNPs and INDELs. SnpEff (v3.1) tool was used to identify synonymous and non-synonymous SNPs, and large-effect SNPs and INDELs (Cingolani et al., 2012). Cut-off for number of non-synonymous SNPs per Kb gene length was determined using Box and Whisker plot to identify the outlier genes.

### Comparative Analysis of PP Variations With a Panel of 108 Rice Lines

We downloaded raw data of 108 selected rice cultivars (Supplementary Table S7) and alignment of this data was performed with reference genome (Os-Nipponbare-Reference-IRGSP-1.0, MSU release 7) using BWA software (v0.7). The combined vcf file was generated using similar parameter and tools. Further, comparative variants analysis was performed to identify the unique variants in PP rice using in-house PERL program.

### Comparative Analysis of PP Variations With Diverse Global Rice Lines Collection

SNP dataset derived from alignment to IRGSP v1.0 was downloaded from SNP-seek database^3^. Corresponding SNPs of PP were filtered as per SNP-seek database norms and compared using Bcftools to determine unique alleles. Identification of different transcripts associated with these variants were also performed. Variations in transcription factor genes were detected using PlantTFDB (Jin et al., 2016). Moreover, comparative analysis of PP genome variations with 3,023 rice genome collections available at Rice SNP-Seek Database was performed (Mansueto et al., 2016).

### Annotation of Variant-Affected Pathways and Traits

All pathways associated with different variants have been annotated using rice metabolic pathway database genes (RiceCyc v3.3) using default parameters (Dharmawardhana et al., 2013). QTLs/Genes morphological, physiological and resistance/tolerance traits were downloaded from Q-TARO database^4^. SNPs in each QTLs/Genes were identified through co-localization of the coordinates in Q-TARO (Yonemaru et al., 2010; Yamamoto et al., 2012).

### Evolutionary Analysis of PP *Bh4* and *Rc* Genes

Search of *Bh4* gene for selected 108 rice lines against various public databases provides only 70 lines with *bh4* genes sequences, while *Rc* gene was present for selected cultivars. Further multiple sequence alignments using ClustalW and phylogenetic tree construction using the Neighbor-Joining method and branch length is estimated using the bootstrap in MEGAv6 (Tamura et al., 2013).

## Accession Number

Whole genome data of PP rice submitted in SRA database with BioProject ID: PRJNA309223.

## Data Availability

The datasets generated for this study can be found in data generated in this work was related to rice plant and submitted in SRA database (Acc No. PRJNA309223).

## Author Contributions

GT, SS, and VL conceived the work and designed the experiments. RG, VL, BK, SL, AMK, NC, and LM performed *in silico* and *in vitro* experiments. RG, VL, GT, and AR analyzed the results. All authors contributed to writing the manuscript, discussed the results, and commented on the manuscript.

## Conflict of Interest Statement

VL, SL, BK, NC, AMK, AR, and GT were employed by AgriGenome Labs Pvt. Ltd., India, SS was employed by SciGenom Labs Pvt. Ltd., India, and RG and LM were employed by MedGenome Labs Ltd., India.

## Footnotes

1 http://qtaro.abr.affrc.go.jp/

2 http://rice.plantbiology.msu.edu/

3 http://snp-seek.irri.org/

4 http://qtaro.abr.affrc.go.jp/
