# Supplementary figures and images for: Whole Genome Sequencing and Comparative Genomic Analysis Reveal Allelic Variations Unique to a Purple Colored Rice Landrace (Oryza sativa ssp. indica cv. Purpleputtu)
Source: Front Plant Sci. 2019 May 7;10:513. doi: 10.3389/fpls.2019.00513 (PMC6516047; doi:10.3389/fpls.2019.00513)

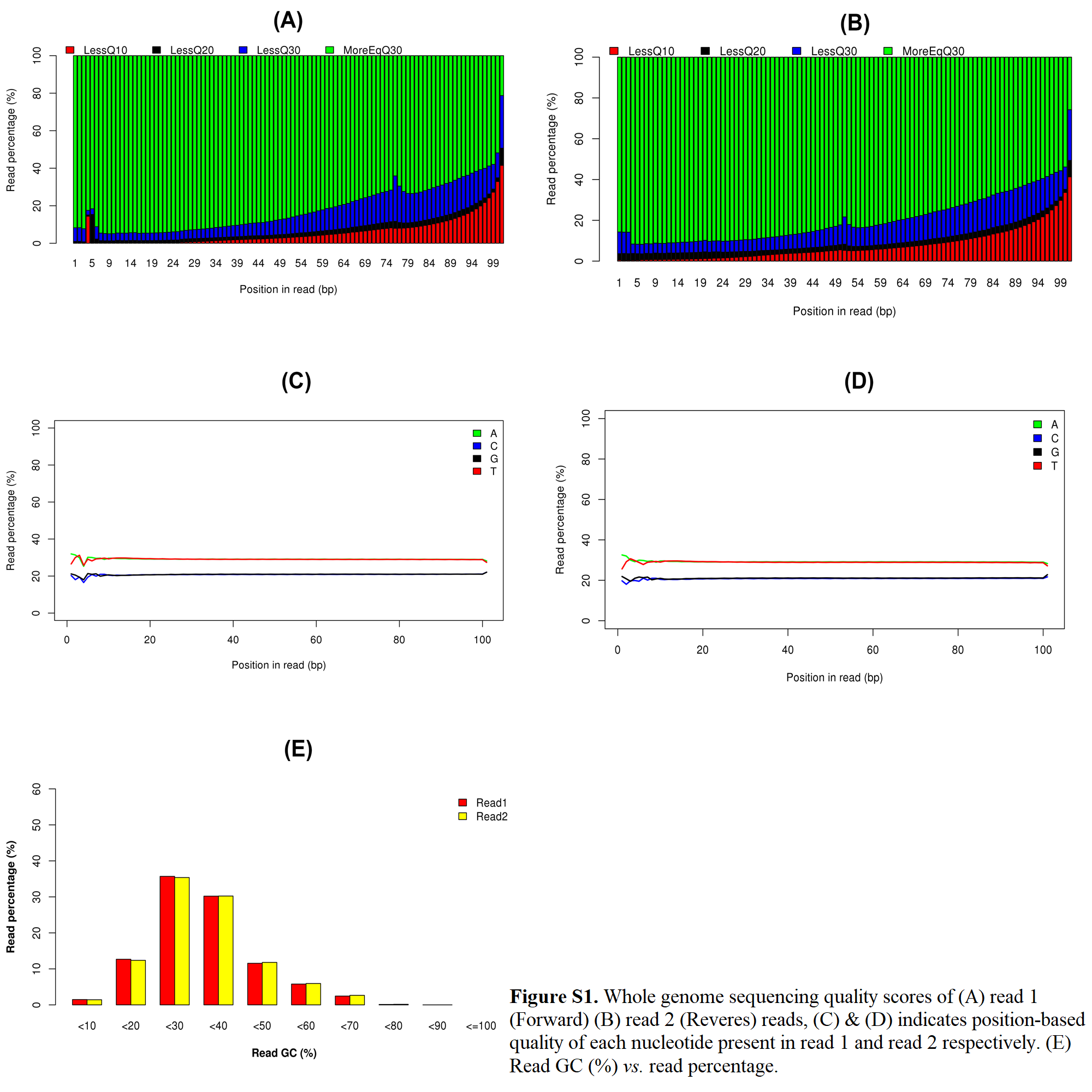

Supplement: Supplementary file 2 [file Image_1.TIF]

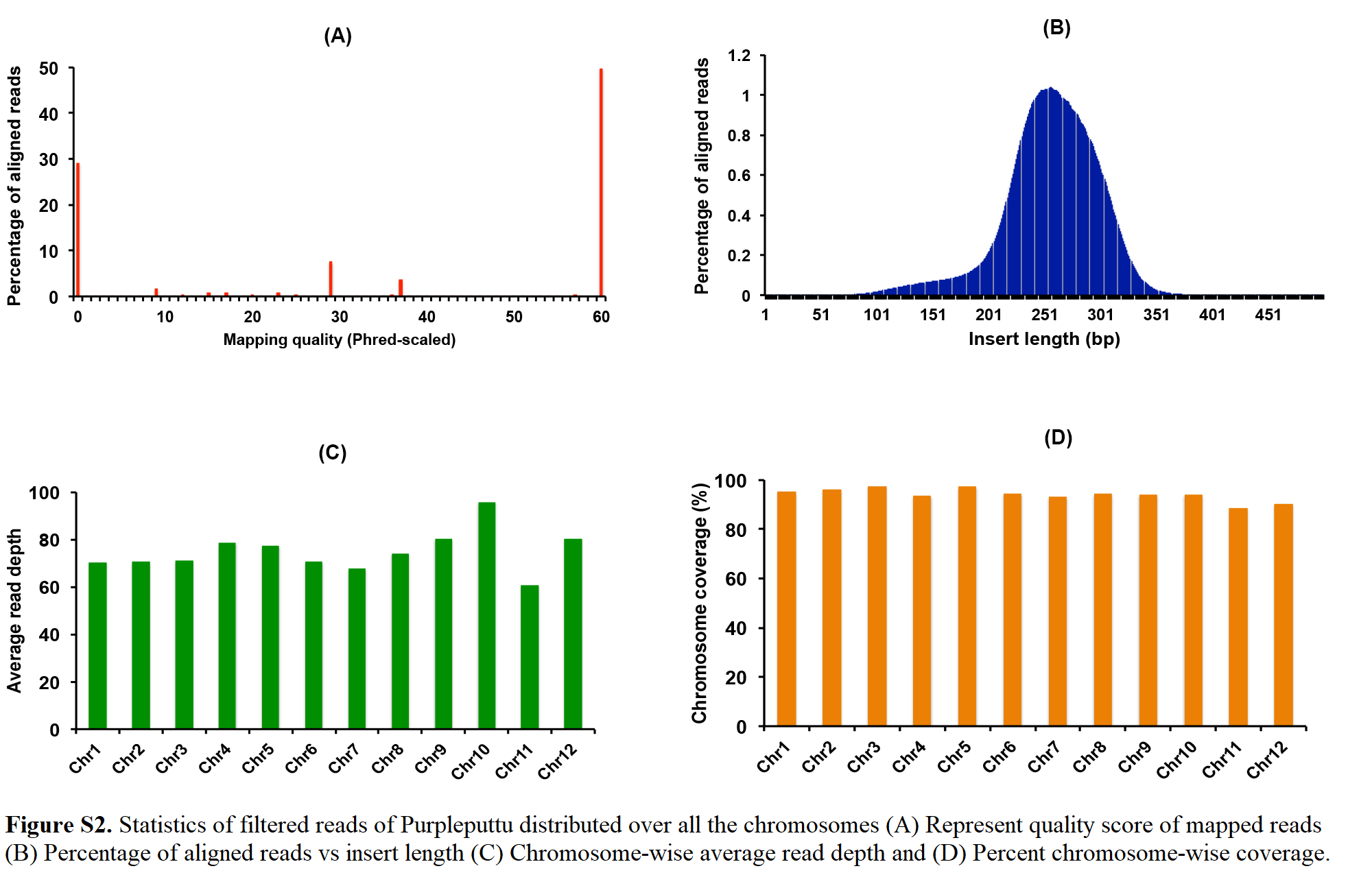

Supplement: Supplementary file 3 [file Image_2.TIF]

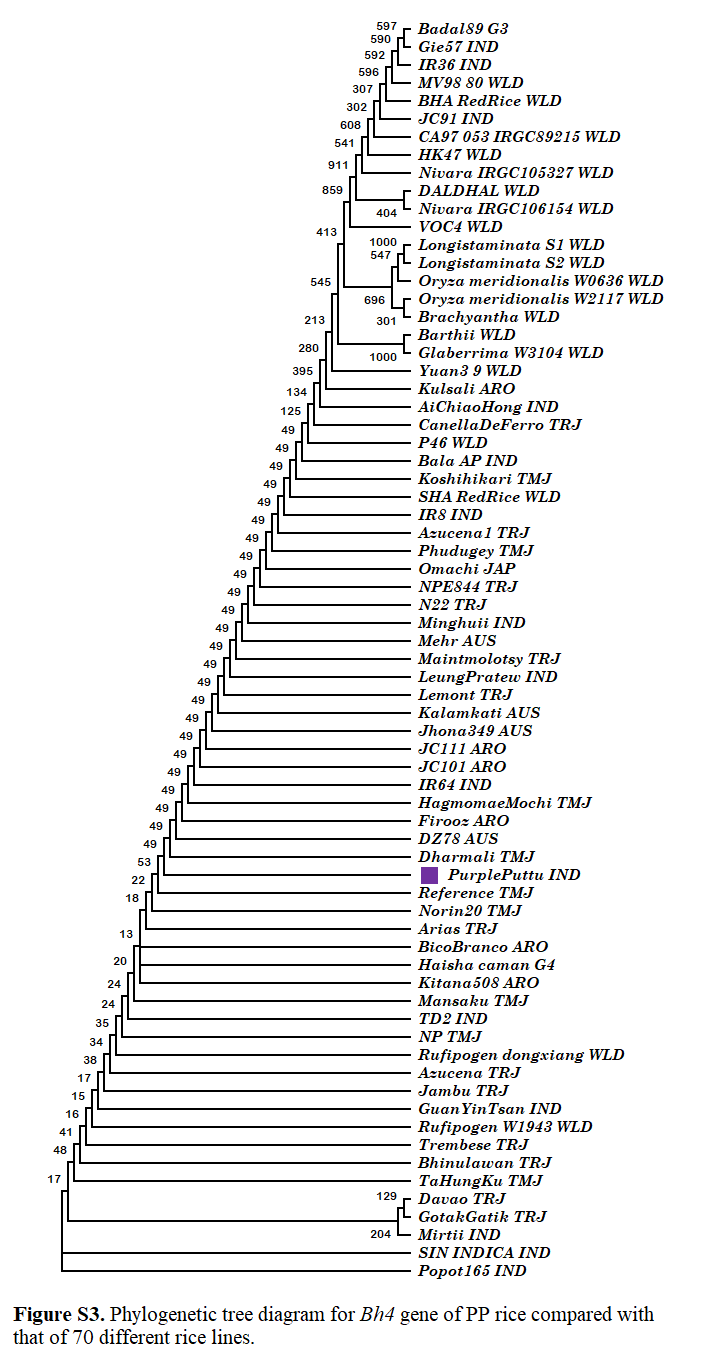

Supplement: Supplementary file 4 [file Image_3.TIF]

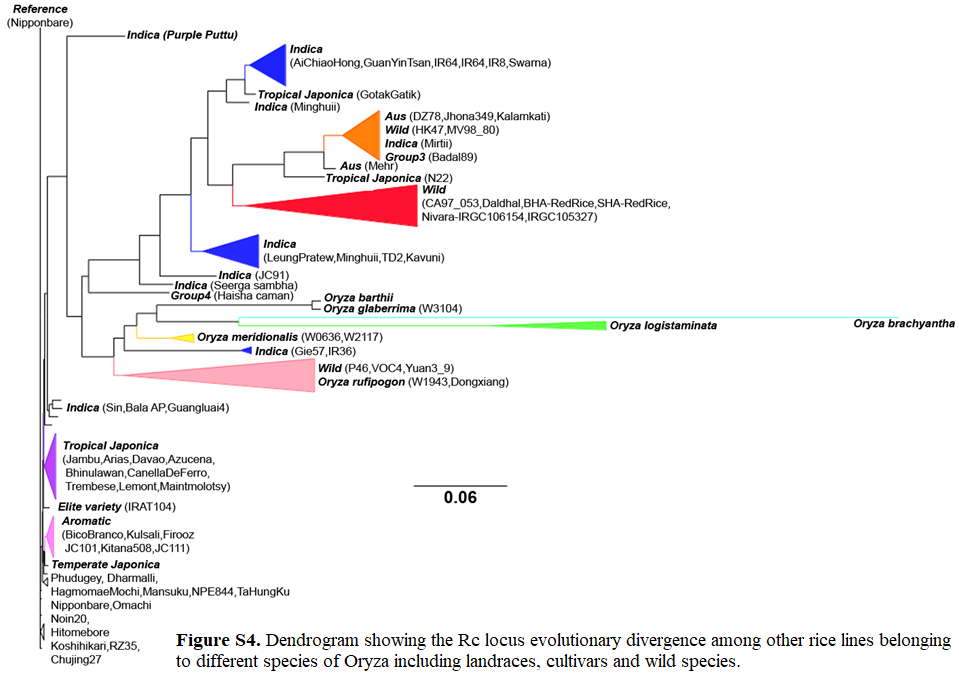

Supplement: Supplementary file 5 [file Image_4.TIF]

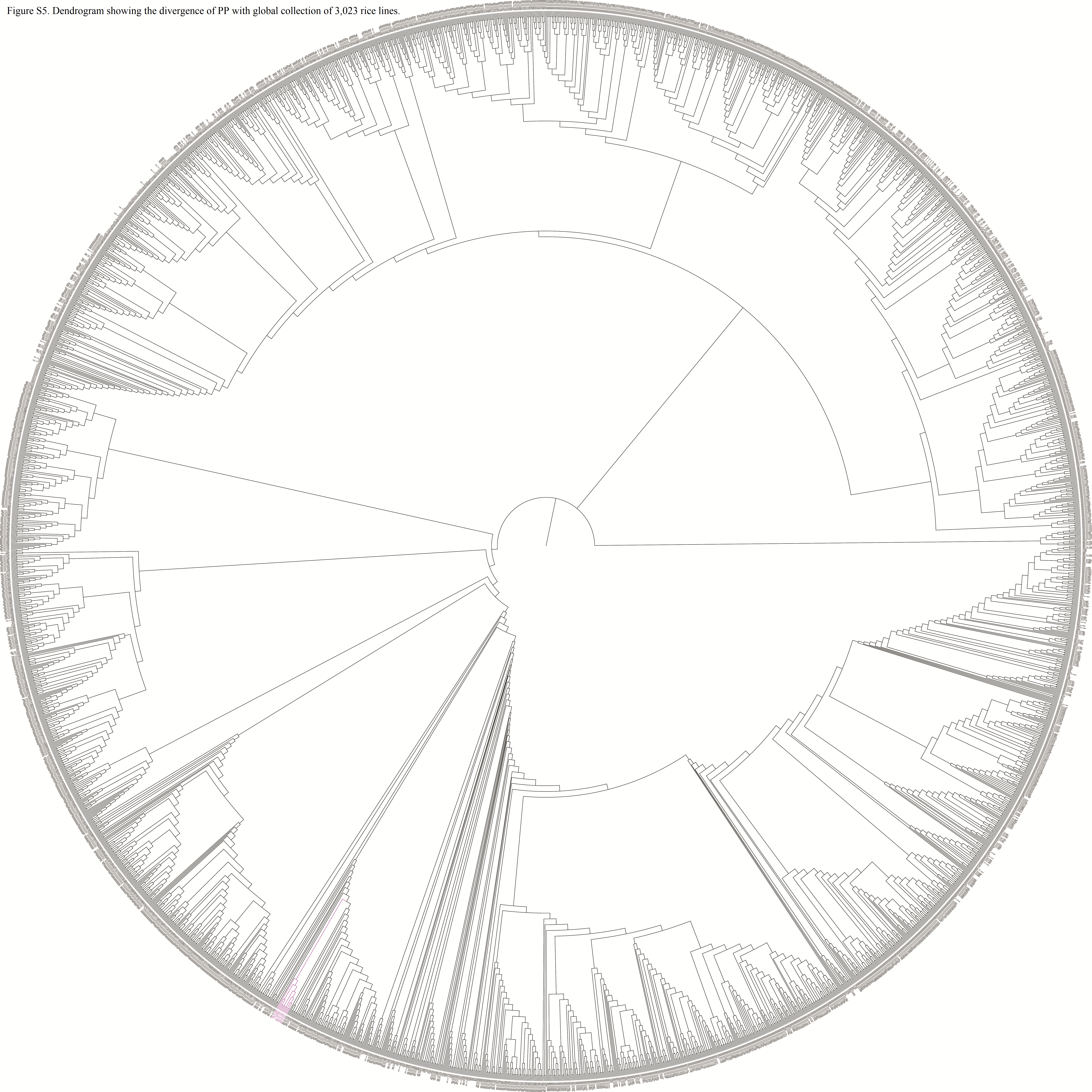

Supplement: Supplementary file 6 [file Image_5.TIF]
